# Robotic urologic applications of the hinotori™ Surgical Robot System

**DOI:** 10.1016/j.ajur.2024.05.002

**Published:** 2024-08-24

**Authors:** Shunsuke Miyamoto, Tomoya Hatayama, Hiroyuki Shikuma, Kazuma Yukihiro, Kyohsuke Iwane, Ryo Tasaka, Yuki Kohada, Takafumi Fukushima, Kenshiro Takemoto, Miki Naito, Kohei Kobatake, Yohei Sekino, Hiroyuki Kitano, Kenichiro Ikeda, Keisuke Goto, Akihiro Goriki, Keisuke Hieda, Nobuyuki Hinata

**Affiliations:** Department of Urology, Graduate School of Biomedical and Health Sciences, Hiroshima University, Hiroshima, Japan

**Keywords:** hinotori™ Surgical Robot System, Surgical robot, Robot-assisted radical cystectomy, Intracorporeal urinary diversion, Robot-assisted radical prostatectomy, Robot-assisted partial nephrectomy, Robot-assisted radical nephrectomy, Robot-assisted nephroureterectomy, Robot-assisted adrenalectomy

## Abstract

**Objective:**

To assess the safety and effectiveness of urological tumor surgeries using the hinotori™ Surgical Robot System (hinotori) in a real-world clinical setting.

**Methods:**

All surgeries including robot-assisted radical prostatectomy (RARP), robot-assisted partial nephrectomy (RAPN), robot-assisted radical nephrectomy (RARN), robot-assisted nephroureterectomy (RANU), robot-assisted adrenalectomy (RAA), and robot-assisted radical cystectomy with intracorporeal urinary diversion (RARC+ICUD) for urological tumors with the hinotori and da Vinci surgical system (da Vinci) from January 2022 to September 2023 were enrolled. We evaluated the safety and effectiveness of surgeries using the hinotori compared with those using the da Vinci.

**Results:**

Robotic surgeries using the hinotori were performed in a total of 91 cases, comprising 42 cases of RARP, 18 cases of RAPN, six cases of RARN, 10 cases of RANU, 13 cases of RAA, and two cases of RARC+ICUD; no major intraoperative complications were observed in any of the cases using the hinotori; no major postoperative complications occurred in any of the cases; no case experienced an unrecoverable equipment error during surgery. Meanwhile, robotic surgeries using the da Vinci were performed in a total of 277 cases, comprising 126 cases of RARP, 94 cases of RAPN, 12 cases of RARN, 10 cases of RANU, 20 cases of RAA, and 15 cases of RARC+ICUD; major intraoperative complications occurred in two cases; major postoperative complications occurred in seven cases; seven cases required transfusion; one case underwent conversion to open surgery; during the study period, no case experienced an unrecoverable equipment error. Surgical outcomes for cases with the hinotori were comparable to those with the da Vinci.

**Conclusion:**

This study demonstrated that the hinotori is a safe and feasible tool for robotic surgeries in the field of urology.

## 1 Introduction

**Figure 1:**
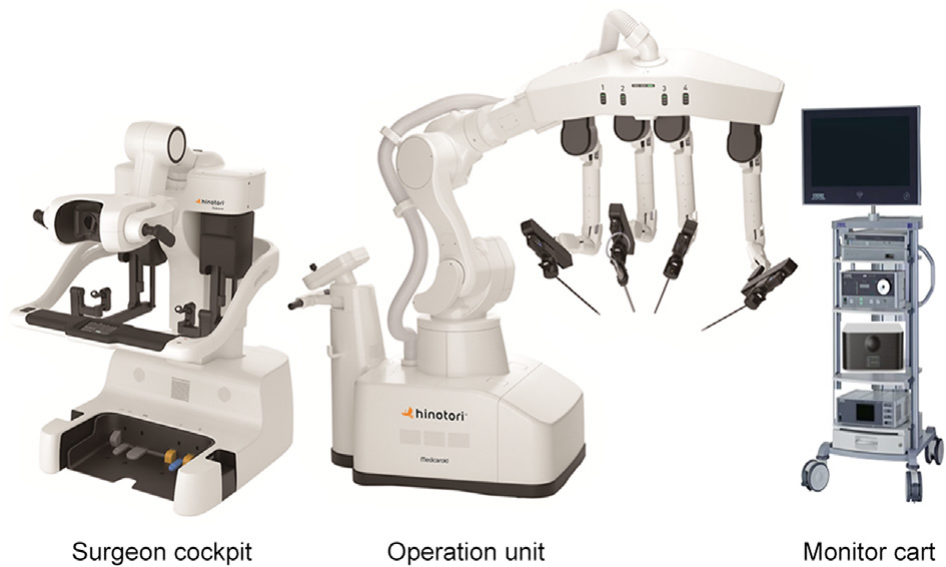
The components of the hinotori™ Surgical Robot System (Medicaroid Corporation, Kobe, Japan).

**Figure 2:**
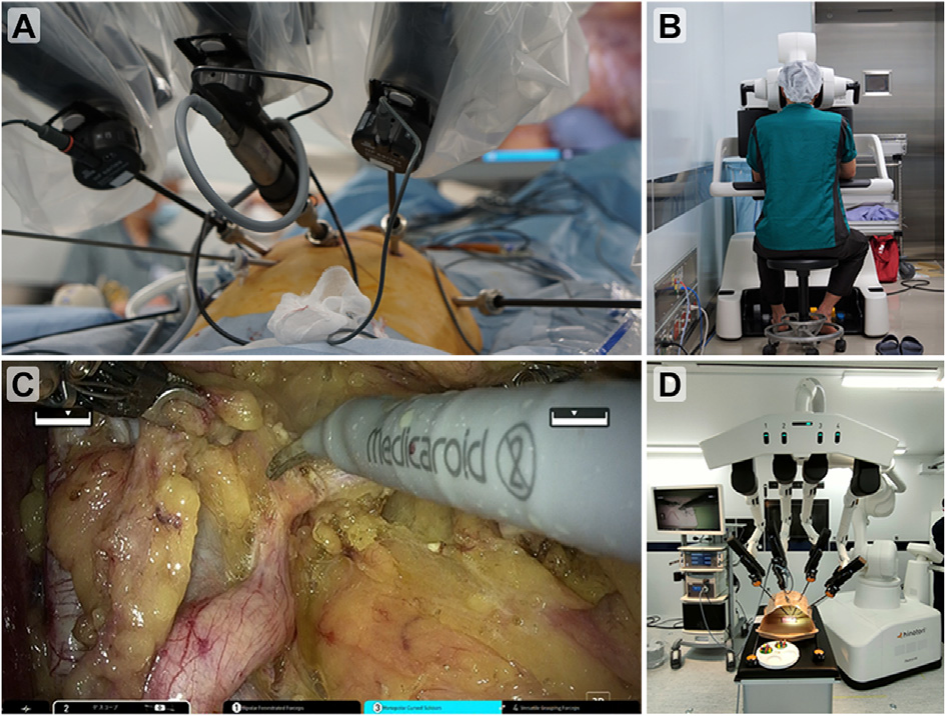
Photos of the hinotori. (A) Patient view of the hinotori wide space available around the trocars; (B) Surgeon cockpit of the hinotori; (C) Monitor image displayed by the hinotori; (D) Simulation using the actual hinotori. hinotori, hinotori^TM^ Surgical Robot System.

The hinotori™ Surgical Robot System (hinotori), is a novel robot-assisted surgical system developed in Japan by Medicaroid Corporation (Kobe, Japan) and jointly funded by Kawasaki Heavy Industries, Ltd. (Kobe, Hyogo, Japan) and Sysmex Corporation (Kobe, Hyogo, Japan). The hinotori used in this study consisted of three units: an operation unit, a surgeon cockpit, and a monitor cart (Fig. 1). Each operating arm has eight axes of freedom and an anti-shake mechanism in which interference between the arms is minimized by computer control. Furthermore, by designing the forceps to allow control without docking the arm and port, a sufficient working area around the port is secured, and extracorporeal interference with the surgeon at the bedside is minimized (Fig. 2A). The hinotori has similar operating methods and instruments to the da Vinci surgical system (da Vinci; Intuitive Surgical Inc., Sunnyvale, CA, USA). The performance of the hinotori during actual surgeries was demonstrated through the video of ileal conduit (Supplementary Video 1). Therefore, in Japan, surgeons holding licenses for the da Vinci are permitted to undergo training in the handling and use of instruments for the hinotori, enabling them to obtain licenses for the hinotori and perform surgeries (Fig. 2). This novel medical device was the first in the world to receive regulatory approval in Japan on August 8, 2020, following which it began to be used in clinical practice in Japan for radical prostatectomy for prostate cancer [1]. The indications for hinotori use have subsequently been expanded, particularly in the field of urology, and as of April 2022, it has been approved for clinical use in urological oncologic surgeries such as prostatectomy, partial nephrectomy, radical nephrectomy, cystectomy, and adrenalectomy in Japan.

The hinotori was introduced at Hiroshima University Hospital in January 2022, and the following surgeries were performed: robot-assisted radical prostatectomy (RARP) in March 2022, robot-assisted radical nephrectomy (RARN) in April 2022, robot-assisted partial nephrectomy (RAPN) in May 2022, robot-assisted radical cystectomy with intracorporeal urinary diversion (RARC+ICUD) and robot-assisted adrenalectomy (RAA) in June 2022, and robot-assisted nephroureterectomy (RANU) in October 2022. We aimed to report our experience with the hinotori in urologic oncologic surgeries and discuss the safety and efficacy of the hinotori.

Supplementary video related to this article can be found at https://doi.org/10.1016/j.ajur.2024.05.002

The following is the supplementary data related to this article:

Multimedia component 2 **Supplementary Video 1** The performance of the hinotori™ Surgical Robot System during intracorporeal urinary diversion with Wallace uretero-enteric anastomosis. Multimedia component 2

## 2 Methods

This research was conducted in accordance with the ethical standards described in the Declaration of Helsinki. The Research Ethics Committee of Hiroshima University approved this study (authorization number: E2022-0018). All procedures had pharmaceutical approval in Japan at the time of this study's commencement. These procedures also had received approval from the Ethics Committee at Hiroshima University and were performed using the da Vinci prior to the current study. Additionally, all surgeons had experienced robot surgeries with the da Vinci and underwent training for both the hinotori and da Vinci with certificates. Therefore, for cases involving use of the hinotori, additional informed consent was waived.

In order to assess the safety and effectiveness of the hinotori in actual clinical practice, surgical outcomes were retrospectively collected and compared with cases performed using the da Vinci during the same period for each procedure including RARP, RAPN, RARN, RANU, RAA, and RARC+ICUD.

### 2.1 Patient selection

All surgeries for urological tumors performed with the hinotori and da Vinci including RARP, RAPN, RARN, RANU, RAA, and RARC+ICUD at Hiroshima University from January 2022 to September 2023 were included. Ninety-one patients underwent surgery with the hinotori, and 277 patients underwent surgery with the da Vinci. The choice on which surgical robot system to use was flexibly determined based on availability. Clinicopathological outcomes and perioperative outcomes were collected retrospectively.

### 2.2 Endpoint

For a comprehensive analysis of safety and efficacy, we compared characteristics including age, body mass index (BMI), sex, American Society of Anesthesiologists (ASA) physical status, and surgical outcomes including intra- and post-operative complications, transfusion rates, conversion to open surgery, and uncoverable equipment error during surgery between the hinotori and da Vinci. Moreover, we compared clinicopathological results, including age, sex, BMI, ASA score, clinical stage, pathological stage, surgical margin, and perioperative outcomes such as surgical access, robotic time (RT), operative time, estimated blood loss, complications, transfusion rates, and conversion rates between the hinotori and da Vinci in each procedure.

### 2.3 Operation

Eleven surgeons certificated by the Japanese Society of Endourology and Robotics as proctors for robotic surgery performed the robotic surgeries with the hinotori. All members of the hinotori surgical team received official training by the Medicaroid Corporation. Our surgical team at Hiroshima University had experience with over 1000 cases of robotic surgery using the da Vinci. Surgery using the hinotori was performed by the same team members as the da Vinci surgical team. Only one surgeon had prior experience with surgery using the hinotori at another facility before the study period, while the other 10 experienced the hinotori for the first time during the study period at our hospital. During the study, three major updates to the arm control system were installed; a hand clutch system was added; and versatile grasping forceps and a clip applier were launched and introduced.

### 2.4 Preoperative clinical data and perioperative outcomes

Preoperative demographics and tumor characteristics were obtained retrospectively from the medical records of this cohort. Intraoperative data, postoperative outcomes, and pathological features were collected. Postoperative complications were retrospectively collected through chart review by a physician 90 days after surgery according to the European Association of Urology Guidelines Panel recommendations on reporting and grading complications [2]. Complications were graded according to the Clavien-Dindo system and were defined as major complications as Grade 3 or higher [3]. Renal function was assessed by an estimated glomerular filtration rate (eGFR) using the serum creatinine level based on the Modification of Diet in Renal Disease equation.

### 2.5 Statistical analysis

The Mann-Whitney *U* test and Chi-squared test were used to evaluate the differences in characteristics, perioperative outcomes, and pathological features between the groups. A *p*-value of <0.05 was considered to indicate statistical significance for each comparison. All statistical analyses were performed using JMP Pro 14.0.0 (SAS Institute, Cary, NC, USA).

## 3 Results

Robotic surgeries using the hinotori were performed in a total of 91 cases, comprising 42 cases of RARP, 18 cases of RAPN, six cases of RARN, 10 cases of RANU, 13 cases of RAA, and two cases of RARC+ICUD; no major intraoperative complications were observed in any of the cases; no case experienced an unrecoverable equipment error during surgery; one case of RARP underwent transfusion. Meanwhile, robotic surgeries using the da Vinci were performed in a total of 277 cases, comprising 126 cases of RARP, 94 cases of RAPN, 12 cases of RARN, 10 cases of RANU, 20 cases of RAA, and 15 cases of RARC+ICUD; major intraoperative complications occurred in two cases of RARP; major postoperative complications occurred in seven cases (three cases of RARP, one case of RAPN, and three cases of RARC); seven cases required transfusion; one case of RARP underwent conversion to open surgery for repairing bowel injury and creating a colostomy; during the study period, no case experienced an unrecoverable equipment error. In surgeries with the hinotori, the proportion of males was significantly lower compared to that with the da Vinci (*p*=0.044). When comparing the hinotori and da Vinci, there were no differences in major complications, transfusion rates, conversion rates to open surgery, or unrecoverable equipment error rates during surgery (Table 1).

**Table 1:** Patient characteristics and surgical outcomes of all cases.

Variable	The hinotori (*n*=91)^a^	The da Vinci (*n*=277)^a^	*p*-Value
Patient characteristic			
Type of procedure			NA
RARP	42	126	
RAPN	18	94	
RARN	6	12	
RANU	10	10	
RAA	13	20	
RARC+ICUD	2	15	
Age, year	68 (60–74)	69 (61–74)	0.6
BMI, kg/m^2^	23.5 (21.9–26.3)	23.4 (21.7–25.7)	0.5
Sex, male	68 (75)	233 (84)	0.044
ASA score			0.5
1–2	85 (93)	252 (91)	
3–4	6 (6.6)	25 (9.0)	
Surgical outcome			
Major intraoperative complication (Clavien-Dindo grade 3 or 4)	0 (0)	2 (0.72)	0.4
Major postoperative complication (Clavien-Dindo grade 3 or 4)	0 (0)	7 (2.5)	0.13
Transfusion	1 (1.1)	7 (2.5)	0.4
Conversion to open surgery	0 (0)	1 (0.36)	0.6
Unrecoverable equipment error during surgery	0 (0)	0 (0)	1

RA, robot-assisted; RARP, RA radical prostatectomy; RAPN, RA partial nephrectomy; RARN, RA radical nephrectomy; RANU, RA nephroureterectomy; RAA, RA adrenalectomy; RARC+ICUD, RA radical cystectomy with intracorporeal urinary diversion; hinotori, hinotori™ Surgical Robot System; da Vinci, da Vinci surgical system; ASA, American Society of Anesthesiologists; BMI, body mass index; NA, not applicable.

a Values are presented as median (interquartile range), *n*, or *n* (%).

### 3.1 RARP

In our hospital, 42 and 126 patients underwent RARP with the hinotori and da Vinci, respectively. Lymph node dissection was performed with the hinotori and da Vinci in 15 and 56 patients, respectively. Patient characteristics are listed in Supplemental Table 1. In the hinotori cohort, median patient age was 68 years; median BMI was 23.4 kg/m^2^; and median prostate-specific antigen level was 7.0 ng/mL with a median prostatic volume of 31.0 mL. The clinical T stage was T1 for 12 patients, T2 for 26 patients, and T3 for four patients.

There were no significant differences between the hinotori and da Vinci in surgical outcomes including RT (median: 170 min and 174 min, respectively), OT (median: 227 min and 239 min, respectively), estimated blood loss (EBL) (median: 137 mL and 131 mL, respectively), major intraoperative complications (0 and 2, respectively), major postoperative complications (0 and 3, respectively), transfusions (1 and 2, respectively), or conversion to open surgery (0 and 1, respectively). No intraoperative complications or conversion to open surgery occurred with the hinotori (Supplemental Table 1). Pathological features are listed in Supplemental Table 1; 10 patients with the hinotori and 26 patients with the da Vinci were pT3. A positive surgical margin was present in seven and 20 patients, and lymph node metastasis occurred in three and eight patients, respectively.

### 3.2 RAPN

Eighteen and 94 patients underwent RAPN with the hinotori and da Vinci, respectively, in our hospital (Supplemental Table 2). The hinotori cohort was older than the da Vinci cohort (median age: 70 years *vs.* 63 years, *p*=0.047). There were no significant differences in BMI, sex, preoperative eGFR, or ASA score between the two groups. Tumor characteristics including the location, tumor diameter, the R.E.N.A.L. nephrometry score, or clinical T stage were not significantly different between the two groups. There were also no significant differences between the hinotori and da Vinci in surgical outcomes including surgical access (transperitoneal access: 13 and 70, respectively), RT (median: 142 min and 165 min, respectively), OT (median: 238 min and 249 min, respectively), ischemia time (median: 21 min and 20 min, respectively), EBL (median: 110 mL and 150 mL, respectively), major postoperative complications (0 and 1, respectively), transfusions (0 and 1, respectively), conversion to open surgery (0 and 0, respectively), or postoperative eGFR (median: 52 mL/min/1.73 m^2^ and 58 mL/min/1.73 m^2^, respectively). No major intraoperative complication or conversion to open surgery occurred (Supplemental Table 2). One case of major postoperative complication and one case with transfusion occurred in the da Vinci group. Pathological features of the patients were listed in Supplemental Table 2. No patient had a positive surgical margin.

### 3.3 RARN

RARN was performed on six and 12 patients with the hinotori and da Vinci, respectively. Patient and tumor characteristics were listed in Supplemental Table 3. There are no significant differences in age, BMI, sex, preoperative eGFR, ASA score, location, tumor diameter, or lymph node dissection between the hinotori and da Vinci. The surgical outcomes were listed in Supplemental Table 3. There were no significant differences between the hinotori and da Vinci in surgical outcomes including surgical access (transperitoneal access: 5 and 11, respectively), RT (median: 110 min and 106 min, respectively), OT (median: 167 min and 190 min, respectively), or EBL (median: 45 mL and 74 mL, respectively). No major intraoperative complications, major postoperative complications, transfusions, or conversion to open surgery occurred. Pathological features of the patients were listed in Supplemental Table 3. No patient had a positive surgical margin.

### 3.4 RANU

RANU was completed in all cases with bladder cuff excision and without conversion to open surgery or repositioning of the patient or port. In the first place, nephrectomy was performed at the kidney direction stage. In the second place, distal ureterectomy and bladder cuff excision were performed at the bladder direction stage. Regional lymphadenectomy was performed for eight and four patients with the hinotori and da Vinci, respectively.

RANU was performed on 10 and 10 patients with the hinotori and da Vinci, respectively. Patient and tumor characteristics were listed in Supplemental Table 4. There were no significant differences in age, BMI, sex, preoperative eGFR, location, or lymph node dissection between the hinotori and da Vinci. The ASA score was significantly lower with the hinotori than the da Vinci. The surgical outcomes were listed in Supplemental Table 4. The median RT (174 min *vs.* 121 min) and OT (237 min *vs.* 188 min) were significantly longer with the hinotori than the da Vinci. There were no significant differences between the hinotori and da Vinci in surgical outcomes including surgical access (transperitoneal access: 8 and 7, respectively) or EBL (median: 50 mL and 75 mL, respectively). No major intraoperative complications, major postoperative complications, or conversion to open surgery occurred. Pathological features of the patients were listed in Supplemental Table 4. No patient had a positive surgical margin.

### 3.5 RAA

Thirteen and 20 patients underwent RAA with the hinotori and da Vinci, respectively. Patient and tumor characteristics were listed in Supplemental Table 5. There are no significant differences in age, BMI, sex, ASA score, or location between the hinotori and da Vinci. The surgical outcomes were listed in Supplemental Table 5. There were no significant differences between the hinotori and da Vinci in surgical outcomes including surgical access (transperitoneal access: 13 and 18, respectively), RT (median: 66 min and 85 min, respectively), OT (median: 133 min and 143 min, respectively), or EBL (median: 7 mL and 23 mL, respectively). No major intraoperative complications, major postoperative complications, transfusions, or conversion to open surgery occurred. There was no patient with a positive surgical margin.

### 3.6 RARC+ICUD

All procedures of RARC were performed by an expert robotic surgeon (Hinata N) who has performed more than 1000 cases of robot surgery. All cases of RARC except one case of the da Vinci that required a shorter OT due to an ASA score of 3 were performed via transperitoneal access, including urethrectomy and lymphadenectomy covering the common iliac, external iliac, internal iliac, obturator, and median sacral lymph nodes. All cases of ICUD were performed using ileal conduit with Wallace uretero-enteric anastomosis. The intestinal anastomosis was reconstructed using Powered ECHELON FLEX (Ethicon, Inc., Cincinnati, OH, USA) in the hinotori, and SureFoam (Intuitive Surgical Inc., Sunnyvale, CA, USA) or Powered ECHELON FLEX in the da Vinci. RARC+ICUD with the hinotori was performed for the first time in the world in two patients.

RARC+ICUD was performed on two and 15 patients with the hinotori and da Vinci, respectively. Patient and tumor characteristics were listed in Supplemental Table 6. There were no significant differences in age, BMI, sex, ASA score, or lymph node dissection between the hinotori and da Vinci. Neoadjuvant chemotherapy was prescribed in two cases with the hinotori and 10 cases with the da Vinci. The surgical outcomes were listed in Supplemental Table 6. There were no significant differences between the hinotori and da Vinci in surgical outcomes including RT (median: 363 min and 349 min, respectively), OT (median: 444 min and 439 min, respectively), or EBL (median: 667 mL and 450 mL, respectively). No major intraoperative complications or conversion to open surgery occurred. Three cases with the major postoperative complication and three cases with transfusion occurred in the da Vinci group. Pathological features of the patients were listed in Supplemental Table 6. There was no patient with a positive surgical margin. Three cases with the da Vinci had lymph node metastasis.

## 4 Discussion

Although the da Vinci has been the dominant player in robot-assisted surgical systems for almost two decades, recently novel surgical robot platforms have been actively developed, and some systems have already been used in real-world clinical settings [[4], [5], [6], [7], [8], [9]]. Among them, the hinotori stands out as the first surgical robotic system developed in Japan [1,10]. The hinotori offers a compact operation arm with eight axes of motion, one more than the da Vinci has. The hinotori has been designed to reduce interference between its arms and interference with the surgeon at the bedside without directly docking the robot arm and trocar. These features have the potential to provide a suitable environment for a wide range of robot-assisted surgical procedures, especially those requiring highly complex surgery [1]. In our subjective experience, during actual surgeries, procedures with the hinotori seemed to have less interference between the arms compared to those with the da Vinci. However, the pattern of interference is different from that of the da Vinci, requiring some adjustment. Furthermore, since the hinotori does not require docking the robot to trocars, it allows for relatively swift undocking. Although not experienced in our study, the potential advantage of reduced undocking time in cases requiring urgent conversion to open surgery due to significant bleeding seems beneficial in terms of patient safety. Additionally, the hinotori's slim arms appeared to result in less interference between the robot and bedside surgeon compared to the da Vinci, particularly when performing concomitant percutaneous procedures such as urethrectomy or urostomy alongside robotic surgery. The precision and operability of the hinotori were deemed comparable to those of the da Vinci. In our study, surgeons with experience in da Vinci surgeries were able to operate the hinotori for the first time without difficulty and successfully complete the surgeries.

In this study, we reported our operational status and surgical results to examine the effectiveness and safety of the hinotori. The safety and efficacy of the hinotori were assessed for the first time in a preclinical study using a living porcine model and fresh cadavers [1] and a first-in-human trial of RARP using the hinotori was performed on 30 patients to evaluate safety profiles [1]. Subsequently, Miyake et al. [10] reported on RAPN, RARN, and RAA. The safety of the hinotori was reported in a prospective observational study of RAPN, followed by a comparison of surgical outcomes with the da Vinci using propensity score matching, which showed that the hinotori had comparable perioperative outcomes to the da Vinci [11]. Additionally, although in a small number of cases, comparable perioperative outcomes were reported between the da Vinci and hinotori for RARN and RAA [12,13]. When comparing the hinotori with the da Vinci in all cases of our cohort, there were no differences observed in major complications, blood transfusion rates, conversion rates to open surgery, or unrecoverable device error rates during surgery, suggesting similar safety profiles. Furthermore, a comparative analysis was conducted by dividing our cases into each surgical procedure. RT and OT were significantly shorter for RANU with the da Vinci, which may be due to omitted lymphadenectomy because of a high ASA score. No differences were found for individual surgeries other than RT and OT for RANU, indicating that the hinotori can be considered efficient and safe for all the described procedures.

The initial study of robot-assisted surgery by the hinotori in humans reported that device errors were detected in 4 of 30 (13%) surgeries [1], likely in relation to updates of the control programs. Throughout the present study period, one device error occurred with the hinotori due to an update of the robotic system, and the surgical plan was changed to laparoscopic surgery before the surgery started. During the surgeries, there were no conversions to either an open or laparoscopic procedure in robotic surgery using the hinotori. In contrast, in robotic surgeries using the da Vinci, conversion or aborting of the surgery due to device failure was reported to occur in 0.4%–0.5% of cases [14,15]. These results suggest that robotic surgery using the hinotori might be at the same level of safety as robotic surgery using the da Vinci in clinical practice.

This study has some limitations. First, this was a retrospective study with a small case series. Additionally, the follow-up period in this study is short. Furthermore, the comparison between the hinotori and da Vinci may be subject to selection bias due to the choice of the surgical system based on availability and differences in surgeons' experience. Therefore, future prospective clinical trials with a large number of patients will be needed to verify the results shown in this study and prove the clinical advantages of the hinotori.

## 5 Conclusion

This study is the first report to comprehensively summarize robotic surgery performed at a single institution using the hinotori, which is approved in the field of urology in Japan. All robotic surgery techniques using the hinotori in the urological surgeries were performed safely and efficaciously.

## Author contributions

*Study concept and design:* Nobuyuki Hinata, Shunsuke Miyamoto.

*Data acquisition:* Shunsuke Miyamoto, Tomoya Hatayama, Hiroyuki Shikuma, Kazuma Yukihiro, Kyohsuke Iwane, Ryo Tasaka, Yuki Kohada, Takafumi Fukushima, Kenshiro Takemoto, Miki Naito, Kohei Kobatake, Yohei Sekino, Hiroyuki Kitano, Kenichiro Ikeda, Keisuke Goto, Akihiro Goriki, Keisuke Hieda, Nobuyuki Hinata.

*Data analysis:* Shunsuke Miyamoto.

*Drafting of manuscript:* Shunsuke Miyamoto, Nobuyuki Hinata.

*Critical revision of the manuscript:* Nobuyuki Hinata.

*Supervision:* Nobuyuki Hinata.

## Conflicts of interest

The authors declare no conflict of interest.
