# Supplementary material for: Robotic urologic applications of the hinotori™ Surgical Robot System
Source: Asian J Urol. 2024 Aug 24;12(2):162–8. doi: 10.1016/j.ajur.2024.05.002 (PMC12126947; doi:10.1016/j.ajur.2024.05.002)
Supplement: Multimedia component 1 [file mmc1.pdf]

Supplemental Table 1 Comparison of patient and tumor characteristics, surgical outcomes, and pathological features between da Vinci and hinotori in RARP.

Variable	The hinotori (*n*=42)[a]	The da Vinci (*n*=126)[a]	*p*-Value
Patient and tumor characteristic			
Age, year	68 (62–74)	70 (64–73)	0.3
BMI, kg/m^2	23.4 (21.8–25.0)	23.6 (22.0–25.0)	0.7
PSA, ng/mL	7.0 (5.1–10.0)	7.2 (5.5–11.4)	0.9
ASA score			0.3
1–2	40 (95)	121 (96)	
3–4	2 (4.8)	5 (4.0)	
ISUP grade group of the prostate biopsy			NA
1	3 (7.1)	14 (11)	
2	15 (36)	29 (23)	
3	10 (24)	32 (25)	
4	9 (21)	37 (29)	
5	5 (12)	14 (11)	
Prostatic volume, mL	31.0 (22.6–36.5)	29.4 (23.6–38.0)	0.6
Clinical T stage			NA
T1	12 (29)	23 (18)	
T2	26 (62)	98 (78)	
T3	4 (9.5)	5 (4.0)	
Lymph node dissection	15 (36)	56 (44)	0.3
Surgical outcome			
Robotic time, min	170 (142–223)	174 (139–222)	1
Operative time, min	227 (199–287)	239 (210–285)	0.7
Estimated blood loss, mL	137 (60–218)	131 (79–236)	0.8
Major intraoperative complication (Clavien-Dindo grade 3 or 4)	0 (0)	2 (1.6)	0.4
Major postoperative complication (Clavien-Dindo grade 3 or 4)	0 (0)	3 (2.4)	0.3
Transfusion	1 (2.4)	2 (1.6)	0.7
Conversion to open surgery	0 (0)	1 (0.79)	0.6
Pathological feature			
Pathological T stage			0.7
T2	32 (76)	100 (79)	
T3	10 (24)	26 (21)	
ISUP grade group of the surgical specimen			NA
1	1 (2.4)	1 (0.79)	
2	17 (40)	50 (40)	
3	18 (43)	51 (40)	
4	2 (4.8)	9 (7.1)	
5	4 (9.5)	15 (12)	
Positive surgical margin	7 (17)	20 (16)	0.9
Lymph node metastasis	3 (7.1)	8 (6.3)	0.9

RARP, robot-assisted radical prostatectomy; hinotori, hinotori™ Surgical Robot System; da Vinci, da Vinci surgical system; ASA, American Society of Anesthesiologists; BMI, body mass index; ISUP, International Society of Urological Pathology; PSA, prostate-specific antigen; NA, not applicable.

[a] Values are presented as median (interquartile range), or *n* (%).

Supplemental Table 2 Comparison of patient and tumor characteristics, surgical outcomes, and pathological features between da Vinci and hinotori in RAPN.

Variable	The hinotori (n=18)[a]	The da Vinci (n=94)[a]	p-Value
Patient and tumor characteristic			
Age, year	70 (63–75)	63 (55–72)	0.047
BMI, kg/m^2	22.7 (22.1–26.2)	24.1 (21.5–26.5)	0.9
Sex, male	10 (56)	67 (71)	0.19
Preoperative eGFR, mL/min/1.73 m^2	64 (48–73)	64 (52–76)	0.7
ASA score			0.2
1–2	18 (100)	87 (93)	
3–4	0 (0)	7 (7.4)	
Location			0.6
Right	6 (33)	37 (39)	
Left	12 (67)	57 (61)	
Tumor diameter, mm	38 (26–51)	30 (23–42)	0.12
R.E.N.A.L. nephrometry score			0.8
4–8	12 (67)	65 (69)	
9–11	6 (33)	29 (31)	
Clinical T stage			0.4
T1a	11 (61)	67 (71)	
T1b	7 (39)	27 (29)	
Surgical outcome			
Surgical access			0.8
Transperitoneal	13 (72)	70 (74)	
Retroperitoneal	5 (28)	24 (26)	
Robotic time, min	142 (121–187)	165 (124–197)	0.17
Operative time, min	238 (200–272)	249 (200–285)	0.12
Ischemia time, min	21 (17–24)	20 (19–22)	0.9
Estimated blood loss, mL	110 (75–205)	150 (70–260)	0.3
Major intraoperative complication (Clavien-Dindo grade 3 or 4)	0 (0)	0 (0)	1
Major postoperative complication (Clavien-Dindo grade 3 or 4)	0 (0)	1 (1.1)	0.7
Transfusion	0 (0)	1 (1.1)	0.7
Conversion to open surgery	0 (0)	0 (0)	1
Postoperative eGFR, mL/min/1.73 m^2	52 (41–65)	58 (45–69)	0.3
Pathological feature			
Tumor characteristics			0.8
Benign	1 (5.6)	7 (7.4)	
Malignant	17 (94)	87 (93)	
Histologic subtype			
Clear cell	15 (88)	74 (85)	
Papillary	0 (0)	5 (5.7)	
Chromophobe	2 (12)	7 (8.0)	
Unclassified	0 (0)	1 (1.1)	
Pathological T stage			
T1a	11 (65)	67 (77)	
T1b	2 (12)	16 (18)	
T2	0 (0)	0 (0)	
T3	4 (24)	4 (4.6)	
Positive surgical margin	0 (0)	0 (0)	1

RAPN, robot-assisted radical nephrectomy; hinotori, hinotori™ Surgical Robot System; da Vinci, da Vinci surgical system; ASA, American Society of Anesthesiologists; BMI, body mass index; eGFR, estimated

glomerular filtration rate.

[a] Values are presented as median (interquartile range), or *n* (%).

Supplemental Table 3 Patient and tumor characteristics, surgical outcomes and pathological features between in RARN.

Variable	The hinotori (*n*=6)[a]	The da Vinci (*n*=12)[a]	*p*-Value
Patients and tumor characteristic			
Age, year	74 (54–82)	72 (63–76)	1
BMI, kg/m^2	22.1（20.9–23.5)	22.7 (19.9–23.2)	0.9
Sex, male	3 (50)	11 (92)	0.083
Preoperative eGFR, mL/min/1.73 m^2	51 (45–64)	49 (37–64)	0.8
ASA score			0.5
1–2	3 (50)	8 (67)	
3–4	3 (50)	4 (33)	
Location			0.5
Right	2 (33)	6 (50)	
Left	4 (67)	6 (50)	
Tumor diameter, mm	56 (28–82)	62 (47–95)	0.4
Clinical T stage			NA
T1	2 (33)	4 (33)	
T2	0 (0)	1 (8.3)	
T3	3 (50)	7 (58)	
T4	1 (17)	0 (0)	
Clinical N stage			0.18
N0	4 (67)	11 (92)	
N1	2 (33)	1 (8.3)	
Clinical M stage			0.3
M0	3 (50)	9 (75)	
M1	3 (50)	3 (25)	
Lymph node dissection	2 (33)	1 (8.3)	0.18
Surgical outcome			
Surgical access			0.6
Transperitoneal	5 (83)	11 (92)	
Retroperitoneal	1 (17)	1 (8.3)	
Robotic time, min	110 (93–111)	106 (74–148)	0.4
Operative time, min	167 (153–177)	190 (132–212)	0.4
Estimated blood loss, mL	45 (23–50)	74 (34–197)	0.086
Major intraoperative complication (Clavien-Dindo grade 3 or 4)	0 (0)	0 (0)	1
Major postoperative complication (Clavien-Dindo grade 3 or 4)	0 (0)	0 (0)	1
Transfusion	0 (0)	0 (0)	1
Conversion to open surgery	0 (0)	0 (0)	1
Pathological feature			
Tumor characteristics			0.5
Benign	0 (0)	1 (8.3)	
Malignant	6 (100)	11 (92)	
Histologic subtypes			NA
Clear cell	5 (83)	9 (82)	
Papillary	1 (17)	1 (9.1)	
Chromophobe	0 (0)	0 (0)	
Others	0 (0)	1 (9.1)	
Pathological T stage			0.6
T1–2	2 (33)	5 (45)	
T3–4	4 (67)	6 (55)	

Positive surgical margin	0 (0)	0 (0)	1
Lymph node metastasis	1 (50)	1 (100)	0.6

RARN, robot-assisted radical nephrectomy; hinotori, hinotori™ Surgical Robot System; da Vinci, da Vinci surgical system; ASA, American Society of Anesthesiologists; BMI, body mass index.

[a] Values are presented as median (interquartile range), or *n* (%).

Supplemental Table 4 Patient and tumor characteristics, surgical outcomes and pathological features in RANU.

Variable	The hinotori (*n*=10)[a]	The da Vinci (*n*=10)[a]	*p*-Value
Patient and tumor characteristic			
Age, year	73 (64–78)	80 (74–85)	0.3
BMI, kg/m^2	21.8 (19.6–23.9)	23.1 (20.1–24.5)	0.5
Sex, male	7 (70)	7 (70)	1
Preoperative eGFR, mL/min/1.73 m^2	65 (48–69)	45 (34–73)	0.19
ASA score			0.025
1–2	10 (100)	6 (60)	
3–4	0 (0)	4 (40)	
Location			0.7
Right	5 (50)	4 (40)	
Left	5 (50)	6 (60)	
Renal pelvic	4 (40)	3 (30)	0.6
Ureteral	6 (60)	7 (70)	
Clinical T stage			NA
Ta	5 (50)	2 (20)	
T1	2 (20)	5 (50)	
T2	0 (0)	3 (30)	
T3	3 (30)	0 (0)	
Clinical N stage			1
N0	0 (0)	0 (0)	
N1	10 (100)	10 (100)	
Clinical M stage			1
M0	0 (0)	0 (0)	
M1	10 (100)	10 (100)	
Lymph node dissection	8 (80)	4 (40)	0.068
Surgical outcome			
Surgical access			0.6
Transperitoneal	8 (80)	7 (70)	
Retroperitoneal	2 (20)	3 (30)	
Robotic time, min	174 (165–185)	121 (93–142)	0.006
Operative time, min	237 (233–260)	188 (160–219)	0.021
Estimated blood loss, mL	50 (22–60)	75 (28–128)	0.3
Major intraoperative complication (Clavien-Dindo grade 3 or 4)	0 (0)	0 (0)	1.
Major postoperative complication (Clavien-Dindo grade 3 or 4)	0 (0)	0 (0)	1
Transfusion	0 (0)	1 (10)	0.3
Conversion to open surgery	0 (0)	0 (0)	1
Pathological feature			
Histologic subtypes			1
Urothelial carcinoma	10 (100)	10 (100)	

Pathological T stage			NA
Ta	4 (40)	1 (10)	
T1	1 (10)	4 (40)	
T2	2 (20)	3 (30)	
T3	3 (30)	2 (20)	
Positive surgical margin	0 (0)	0 (0)	1
Lymph node metastasis	0 (0)	1 (10)	0.3

RANU, robot-assisted nephroureterectomy; hinotori, hinotori™ Surgical Robot System; da Vinci, da Vinci surgical system; ASA, American Society of Anesthesiologists; BMI, body mass index; NA, not applicable.

[a] Values are presented as median (interquartile range), or *n* (%).

Supplemental Table 5 Patient and tumor characteristics, surgical outcomes and pathological features in RAA.

Variable	The hinotori (*n*=13)[a]	The da Vinci (*n*=20)[a]	*p*-Value
Patient and tumor characteristic			
Age, year	56 (30–60)	54 (40–64)	0.7
BMI, kg/m^2	24.9 (22.8–29.4)	26.3 (23.9–28.8)	0.7
Sex, male	4 (31)	13 (65)	0.055
ASA score			0.3
1–2	12 (92)	16 (80)	
3–4	1 (7.7)	4 (20)	
Location, right/left			0.092
Right	3 (23)	11 (55)	
Left	10 (77)	9 (45)	
Diagnosis			NA
Primary aldosteronism	10 (77)	11 (55)	
Cushing syndrome	2 (15)	4 (20)	
Pheochromocytoma	1 (7.7)	5 (25)	
Surgical outcome			
Surgical access			0.2
Transperitoneal	13 (100)	18 (90)	
Retroperitoneal	0 (0)	2 (10)	
Robotic time, min	66 (37–89)	85 (71–116)	0.085
Operative time, min	133 (111–164)	143 (128–176)	0.2
Estimated blood loss, mL	7 (3–15)	23 (5–49)	0.14
Major intraoperative complication (Clavien-Dindo grade 3 or 4)	0 (0)	0 (0)	1
Major postoperative complication (Clavien-Dindo grade 3 or 4)	0 (0)	0 (0)	1
Transfusion	0 (0)	0 (0)	1
Conversion to open surgery	0 (0)	0 (0)	1
Positive surgical margin	0 (0)	0 (0)	1

RAA, robot-assisted adrenalectomy; hinotori, hinotori™ Surgical Robot System; da Vinci, da Vinci surgical system; ASA, American Society of Anesthesiologists; BMI, body mass index; NA, not applicable.

[a] Values are presented as median (interquartile range), or *n* (%).

Supplemental Table 6 Patient and tumor characteristics, surgical outcomes and pathological features in RARC+ICUD.

Variable	The hinotori (n=2)[a]	The da Vinci (n=15)[a]	p-Value
Patient and tumor characteristic			
Age, year	75 (71–79)	71 (64–79)	0.4
BMI, kg/m^2	21.8 (21.3–22.3)	23.7 (21.9–25.7)	0.057
Sex, male	2 (100)	9 (60)	0.3
ASA score			0.7
1–2	2 (100)	14 (93)	
3–4	0 (0)	1 (6.7)	
Clinical T stage			NA
T1	0 (0)	1 (6.7)	
T2	2 (100)	8 (53)	
T3	0 (0)	5 (33)	
T4	0 (0)	1 (6.7)	
Clinical N stage			0.074
N0	1 (50)	14 (93)	
N1	1 (50)	1 (6.7)	
Clinical M stage			1
M0	2 (100)	15 (100)	
M1	0 (0)	0 (0)	
Neoadjuvant chemotherapy	2 (100)	10 (67)	0.3
Surgical outcome			
Robotic time, min	363 (329–396)	349 (322–391)	1
Operative time, min	444 (395–492)	439 (396–482)	0.9
Estimated blood loss, mL	667 (562–771)	450 (312–974)	0.7
Major intraoperative complication (Clavien–Dindo 3 or 4)	0 (0)	0 (0)	1
Major postoperative complication (Clavien–Dindo 3 or 4)	0 (0)	3 (20)	0.5
Lymph node dissection	2 (100)	14 (93)	0.7
Urethral dissection	2 (100)	14 (93)	0.7
Transfusion	0 (0)	3 (20)	0.5
Conversion to open surgery	0 (0)	0 (0)	1
Pathological feature			
Histologic subtype			0.7
Urotherial carcinoma	2 (100)	14 (93)	
Squamous cell carcinoma	0 (0)	1 (6.7)	
Pathological T stage			NA
T0	2 (100)	3 (21)	
Ta, T1	0 (0)	3 (21)	
T2–T4	0 (0)	8 (57)	
Positive surgical margin	0 (0)	0 (0)	1
Lymph node metastasis			0.5
N0	2 (100)	11 (79)	
N1–2	0 (0)	3 (21)	

RARC+ICUD, robot-assisted radical cystectomy with intracorporeal urinary diversion; hinotori, hinotori™ Surgical Robot System; da Vinci, da Vinci surgical system; ASA, American Society of Anesthesiologists; BMI, body mass index; NA, not applicable.

[a] Values are presented as median (interquartile range), or n (%).
